# Effects of KLK Peptide on Adjuvanticity of Different ODN Sequences

**DOI:** 10.3390/vaccines4020014

**Published:** 2016-05-04

**Authors:** Ghania Chikh, Rachel Luu, Shobhna Patel, Heather L. Davis, Risini D. Weeratna

**Affiliations:** Pfizer Vaccine Immunotherapeutics, Ottawa Laboratories, Ottawa, ON K2K 3A2, Canada; gchikh@engeneinc.com (G.C.); rluu@arbutusbio.com (R.L.); shobhna.patel@abbott.com (S.P.); heather.davis@seqirus.com (H.L.D.)

**Keywords:** vaccine, adjuvant, antimicrobial peptides, TLR9, CpG ODN

## Abstract

Endosomal Toll-like receptors (TLR) such as TLR3, 7, 8 and 9 recognize pathogen associated nucleic acids. While DNA sequence does influence degree of binding to and activation of TLR9, it also appears to influence the ability of the ligand to reach the intracellular endosomal compartment. The KLK (KLKL5KLK) antimicrobial peptide, which is immunostimulatory itself, can translocate into cells without cell membrane permeabilization and thus can be used for endosomal delivery of TLR agonists, as has been shown with the IC31 formulation that contains an oligodeoxynucleotide (ODN) TLR9 agonist. We evaluated the adjuvant activity of KLK combined with CpG or non-CpG (GpC) ODN synthesized with nuclease resistant phosphorothioate (S) or native phosphodiester (O) backbones with ovalbumin (OVA) antigen in mice. As single adjuvants, CpG(S) gave the strongest enhancement of OVA-specific immunity and the addition of KLK provided no benefit and was actually detrimental for some readouts. In contrast, KLK enhanced the adjuvant effects of CpG(O) and to a lesser extent of GpC (S), which on their own had little or no activity. Indeed while CD8 T cells, IFN-γ secretion and humoral response to vaccine antigen were enhanced when CpG(O) was combined with KLK, only IFN-γ secretion was enhanced when GpC (S) was combined to KLK. The synergistic adjuvant effects with KLK/ODN combinations were TLR9-mediated since they did not occur in TLR9 knock-out mice. We hypothesize that a nuclease resistant ODN with CpG motifs has its own mechanism for entering cells to reach the endosome. For ODN without CpG motifs, KLK appears to provide an alternate mechanism for accessing the endosome, where it can activate TLR9, albeit with lower potency than a CpG ODN. For nuclease sensitive (O) backbone ODN, KLK may also provide protection from nucleases in the tissues.

## 1. Introduction

Endosomal Toll-like receptors (TLR) such as TLR3, 7, 8 and 9 recognize pathogen associated nucleic acids. TLR9 normally recognizes pathogen-associated DNA, but can also be activated by synthetic oligodeoxynucleotides (ODN), with length, sequence, backbone and formulation all contributing to the degree of activation. While DNA sequence does influence the degree of binding to and activation of TLR9 in itself, it also seems to influence the ability of the ODN to reach the intracellular endosomal compartment in order to interact with TLR9 [1,2,3].

Anti-microbial peptides, also known as host defense peptides, possess a broad spectrum of antimicrobial properties. Some of these membrane active compounds can translocate into cells without cell membrane permeabilization and hence have potential use for intracellular drug delivery [4]. KLK (KLKL5KLK), a synthetic poly-cationic peptide, has immunomodulatory properties including activation of human neutrophils and U937 monocytes to produce superoxide anions through binding to cell surface calreticulin [5,6]. When combined with an antigen, KLK has been shown to increase antigen delivery to antigen presenting cells (APC) and promote Th2 biased antigen-specific immune responses [7]. The adjuvant system IC31® (Valneva, originally from Intercell AG, Lyon, France) comprises of KLK combined with an oligonucleotide (ODN) of repeating deoxyinosine and deoxycytosine dinucleotides (polyI:C) with a natural phosphodiester backbone (ODN1a) and has been shown to augment immune responses against various antigens in pre-clinical models [8,9,10,11,12,13,14,15,16]. In clinical testing, IC31® elicited strong and long lasting T cell responses and antigen-specific IgG responses against mycobacterial antigens 85B, ESAT-6 and Rv2660c in both healthy volunteers and TB infected individuals. Antigen specific T cell responses included IFNγ secreting T cells measured by enzyme-linked immunospot (ELISpot) assay, poly-functional CD4 T cells expressing IFN-γ, TNF-α and IL-2 and enhanced CD4 central memory T cells [15,17].

Synthetic ODN containing immunostimulatory CpG motifs (CpG) activate immune cells through a toll-like receptor 9 (TLR9) and are potent adjuvants with a Th1-bias, even when combined with Th2 adjuvants such as alum [18,19]. CpG are also effective adjuvants in humans, especially when used in combination with aluminum hydroxide (alum) [20,21,22]. For *in vivo* adjuvant use, CpG are often manufactured with a phosphorothioate (S) nuclease-resistant backbone, especially if they are to be used in a formulation that exposes them to extracellular environment. CpG with the nuclease sensitive native phosphodiester (O) backbone are usually inactive unless protected through formulation, for example in liposomes or microparticles [1,23,24]. ODN without CpG motifs, such as that used in the IC31® adjuvant system, also act in a TLR9-dependent fashion to promote Th1 biased immune responses, although they are less potent than CpG ODN for TLR9 activation, so typically higher doses are required and even then, responses are often weaker [25].

The current study evaluated the adjuvant effects of KLK with CpG or non-CpG of the same sequence except with CG to GC dinucleotide reversal (GpC); both sequences were evaluated with nuclease sensitive and nuclease resistant backbones.

## 2. Materials and Methods

### 2.1. Animals

Wild type (WT) C57BL/6 mice (Charles River Laboratories, Montreal, QC, Canada) and TLR9 knock-out mice (TLR9 KO) back-crossed to C57BL/6 for eleven generations as previously described [26] (Courtesy of Dr. Shizuo Akira, Osaka University, Japan), were housed in ventilated micro-isolator cages and provided food and water *ad libitum*. All procedures performed on animals in this study were in accordance with regulations and guidelines reviewed and approved by the Pfizer Institutional Animal Care and Use Committee and were conducted in facilities fully accredited by AAALAC International.

### 2.2. Adjuvants

KLK peptide (KLKL5KLK) and KPK peptide (KPKP5KPK) were synthesized by CPC Scientific, San Jose, CA, USA.

All ODN sequences were derived from CpG 1826, a murine optimized B-Class CpG ODN that has been used widely in published studies [27]. CpG 1826 is normally synthesized with a nuclease-resistant phosphorothioate backbone, which hereafter will be referred to as CpG(S). The same sequence with a native phosphodiester backbone is referred to as CpG(O). Additionally two non-CpG ODN were made by reversing the CG to a GC to make GpC(S) and CpG(O). All ODN, including the fluorescein amidite (FAM) labelled CpG(S) were manufactured by TriLink Biotechnologies (San Diego, CA, USA). Table 1 summarizes the ODN sequences and backbones.

### 2.3. Immunization and Sample Collection

Mice received OVA antigen (10 μg, Sigma-Aldrich, Oakville, ON, Canada) alone or in combination with ODN (4 nmol) and/or KLK or KPK peptides (100 nmol). KPK peptides were used only in studies with WT mice. Vaccines were formulated in sorbitol buffer (0.27 M sorbitol, 10 mM Tris, pH 7.4. The doses of antigen, KLK/KPK peptides, ODN and formulation buffer were based on previously published work [28,29]. Vaccine formulations were administered in a total volume of 50 μL by intramuscular (IM) injection into the tibialis anterior muscle of mice at weeks 0, 2 and 3. Plasma and splenocytes were collected at 1 week after the last dose for assessment of antigen-specific immune responses.

### 2.4. Adaptive Immune Responses

OVA-specific antibody (Ab) responses were quantified in plasma recovered from individual animals using end point ELISA (in triplicate) as previously described [19], except ELISA plates were coated with 100 µL of 10 μg/mL OVA in 0.05 M sodium carbonate-bicarbonate buffer, pH 9.6.

OVA-specific CD8 T cells were evaluated by staining splenocytes with rat anti-mouse monoclonal Ab: CD8-FITC (clone 53-6.7), CD4-PerCP (clone RM4-5) and CD3-FITC (clone 17A2) (Becton Dickinson, Mississauga, ON, Canada), LIVE/DEAD®Fixable Aqua Dead Cell stain (Invitrogen, New York, NY, USA) and iTAg MHC Tetramer SIINFEKL/H-2Kb-PE (Beckman Coulter Immunomics, Palatine, IL, USA). Samples were acquired using BD FACSCanto II and analyzed with FlowJo software (Tree Star, Inc., Ashland, OR, USA).

OVA-specific cytokine secretion was measured using splenocytes. Briefly, splenocytes were prepared and adjusted to a final concentration of 5 × 10^6^ cells per mL in RPMI 1640 (Hyclone, Logan, UT, USA) supplemented with 2% normal mouse serum (Cedarlane Laboratories, Burlington, ON, Canada), 1% Penicillin/Streptomycin (Invitrogen), 1% L-Glutamine (Invitrogen) and 0.005 mM β-mercaptoethanol (Invitrogen). Cells were plated onto 96-well U-bottom plates (100 μL/well) along with 100 μL of each stimulant and incubated in a humidified 5% CO_2_ incubator at 37 °C for 24 h (for IL-2 and IL-4) and 72 h (for IFN-γ Culture, supernatants were harvested and stored at −80 °C until assayed. Commercially available assay kits (OptEIA kits) for detection of mouse IFN-γ, IL-2 and IL-4; (BD Pharmingen, San Jose, CA, USA) were used to assay cytokine levels.

### 2.5. Innate Immune Responses

Other mice received a single IM administration (as for above vaccine studies) of unadjuvanted OVA (10 μg) or OVA in combination with ODN (4 nmol) alone, or with KLK or KPK peptide (100 nmol). Plasma and spleens were collected at 24 h post injection.

Plasma cytokines were measured using the ELISA kits mentioned above. Splenic cellular activation was evaluated by multi-color flow cytometry using fluorescent conjugated monoclonal Ab (Becton Dickinson, Mississauga, ON, Canada). The monoclonal Ab panel included cell surface markers B220 (anti-B220-APC-Cy7; clone RA3-6B2), CD11b (anti-CD11b-PE-Cy7; clone M1/70), MHCII (anti-MHCII-BV421; clone M5/114), cell activation markers CD86 (anti-CD86-APC; clone GL1) and CD80 (anti-CD80-PE; clone 16-10A1). LIVE/DEAD®Fixable Aqua Dead Cell (Invitrogen) was used to exclude dead cells and samples were acquired using BD FACSCanto II and data were analyzed with FlowJo software (Tree Star).

Uptake of CpG(S) by APC in the draining lymph nodes was assessed in WT C57BL/6 mice (2/group). Mice were injected IM with 4 nmol CpG(S) alone or in combination with KLK (100 nmol). Control mice were injected with 50 μL of Sorbitol buffer. Then, at 5, 24 and 48 h, draining lymph nodes were removed and ODN uptake by APC was assessed by multi-color flow cytometry using anti-CD11b-PE-Cy7 (clone M1/70) and anti-CD11c-PerCP-Cy5.5 (clone HL3, Becton Dickinson). LIVE/DEAD®Fixable Aqua Dead Cell Stain was used to exclude dead cells and samples were acquired using BD FACSCanto II and data were analyzed with FlowJo software.

### 2.6. Statistical Analysis

Data were analyzed using GraphPad Prism (GraphPad Software, San Diego, CA, USA). Statistical significance of the difference between two groups was calculated by Student’s 2-tailed *t*-test and between three or more groups by 1-factor analysis of variance (ANOVA), followed by post-hoc analysis using either Dunnett’s (comparison with control group) or Tukey’s (comparison between groups) multiple comparison tests. Differences were considered to be not significant with *p* > 0.05.

## 3. Results

### 3.1. Humoral Immunity

Adjuvant effects of the various single agent or combination formulations on anti-OVA Ab responses are summarized in Table 2.

**Figure 1:**
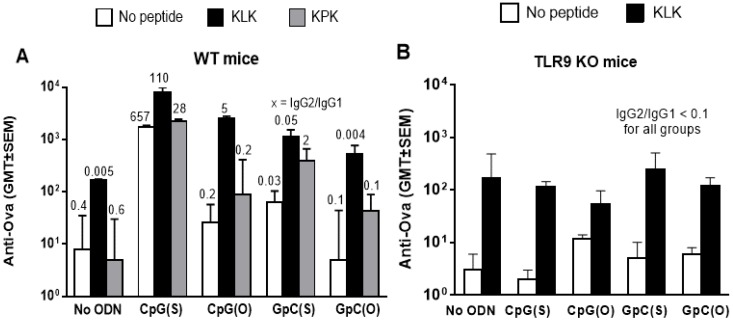
Wild type C57Bl/6 (Panel A) or TLR9 deficient (Panel B) mice (*n* = 5/group) were immunized at week 0, 2 and 3 by IM injection with unadjuvanted OVA (10 μg) or OVA adjuvanted with CpG(S), CpG(O), GpC(S), GpC(O) ODN (4 nmol) alone or in combination with KLK or KPK (only WT mice) peptides (100 nmol). Each bar represents the group geometric mean (±SEM) of the titer for OVA-specific antibodies (anti-OVA IgG) in plasma taken 1 week after final immunization. Numbers above each bar indicate the IgG2c/IgG1 isotype ratio.

In WT mice, both CpG(S) and KLK as sole adjuvants were capable of significantly enhancing OVA-specific Ab titers over non-adjuvanted OVA (*p* ≤ 0.0001 and 0.001, respectively) and CpG(S) alone was superior to KLK alone (*p* ≤ 0.01). Furthermore, the combination of CpG(S) with KLK gave better Ab responses than KLK alone (*p* ≤ 0.0001) but not better than CpG(S) alone (*p* > 0.05), indicating a lack of additive or synergistic effects between KLK and this ODN which has an optimal backbone and sequence for TLR9 activation (Figure 1A).

As expected, the nuclease sensitive CpG(O) and the less potent TLR9 activating GpC (in either backbone) were ineffective as adjuvants on their own since anti-OVA titers were not greater than those with non-adjuvanted OVA (*p* > 0.05). When combined with KLK, CpG(O) induced significantly greater anti-OVA Ab titers compared to KLK alone (*p* ≤ 0.01). This is likely through KLK-mediated protection of CpG(O) from nuclease degradation and enhanced cellular uptake of this ODN. However, when GpC (in either backbone) were combined with KLK, anti-OVA titers were greater than unadjuvanted OVA (*p* ≤ 0.01) but not greater than KLK alone (*p* > 0.05) indicating that KLK could not overcome the lack of optimal immunostimulatory motifs of these ODN, at least for effect on Ab titer (Figure 1A).

The KPK control peptide had no adjuvant activity on Ab titers when used alone (*p* > 0.05 compared to OVA alone). Nor did any KPK/ODN combination induce higher Ab titers than that ODN alone (*p* > 0.05), indicating the synergistic effects of KLK with CpG(O) and GpC(S) were specific to KLK *versus* being a non-specific ODN-peptide interaction (Figure 1A).

IgG2c/IgG1 ratios were used as a surrogate of the T-helper (Th) bias of the response. For the two adjuvants that worked on their own, CpG(S) promoted a strong Th1 biased immune responses (high ratio) whereas KLK resulted in the default Th2 biased immune response to OVA (low ratio). In combination with KLK, CpG(S) dominated for Th bias since strong Th1 responses were maintained. Interestingly, KLK with CpG(O) was also Th1, indicating that once KLK overcame the deficiency of the unstable backbone, the CpG motif could exert its effect on the TLR9 (Figure 1A).

Anti-OVA Ab titers in TLR9−/− mice were similar to those in WT mice with OVA alone or OVA plus KLK, which was expected since neither the OVA antigen nor KLK peptide involve TLR9 activation. However, those ODN formulations that enhanced responses in WT mice, namely CpG(S), KLK/CpG(S) and KLK/CpG(O), all failed to do so in TLR9 KO mice (*p* > 0.04 versus OVA alone), indicating that any CpG adjuvant effect was TLR9 mediated. (Figure 1B).

### 3.2. Cellular Immunity

**Figure 2:**
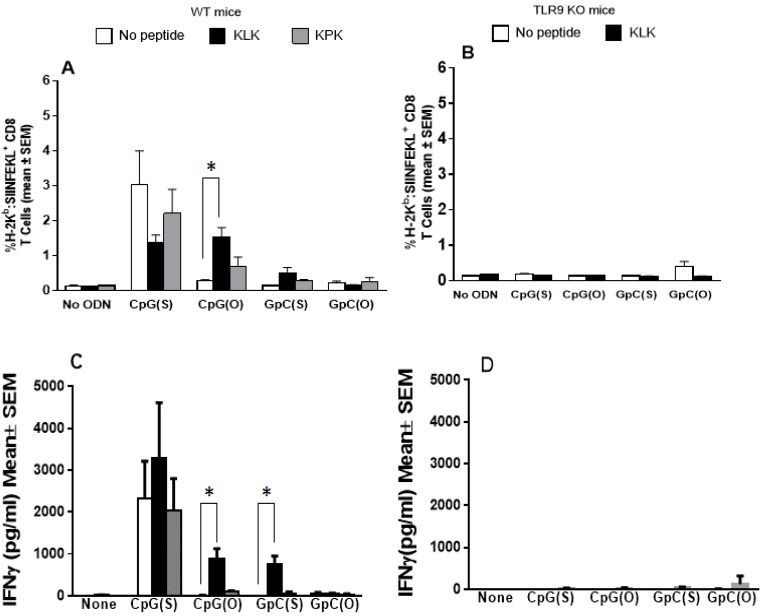
Wild type C57Bl/6 (Panel A & C) or TLR9 deficient (Panel B & D) mice (*n* = 5/group) were immunized at week 0, 2 and 3 by IM injection with unadjuvanted OVA (10 μg) or OVA adjuvanted with CpG(S), CpG(O), GpC(S), GpC(O) ODN (4 nmol) alone or in combination with KLK or KPK (only WT mice) peptides (100 nmol). (**A**,**B**): Splenocytes collected 1 week after final immunization were used to determine the percentage of OVA tetramer positive CD8 populations as measured by FACS. Bars represent the group mean (±SEM) for the % OVA-specific CD8 T cell population within the total splenic CD8 T cells; (**C**,**D**): Splenocytes collected one week after final immunization were re-stimulated with 0.5 mg/mL of OVA for 72 h. Level of IFNγ in culture supernatants were determined by ELISA. Bars represent the mean cytokine concentration (pg/mL) ± the standard error. Significant differences between groups is shown by * where * = *p* < 0.05.

In WT mice, no or only very low numbers of OVA-specific CD8 T cells were induced with non-adjuvanted OVA. As a sole adjuvant, only CpG(S) significantly enhanced numbers of CD8 T cells compared to all other groups (*p* ≤ 0.001), with no enhancement over OVA alone for the other ODN, KLK or KPK (*p* > 0.05). (Figure 2A). There was no benefit to combining the CpG(S) with KLK (*p* > 0.05 compared to CpG(S) alone) and indeed, there was a trend to poorer responses with a lower number of OVA-specific CD8 T cells being observed in 4 out of 5 animals immunized with the combination compared to CpG(S) alone. In contrast, the combination of KLK/CpG(O) did augment OVA-specific CD8 T cells over OVA alone or with either adjuvant alone (*p* ≤ 0.001), indicating that, as seen with the Ab response, the KLK helped the ODN overcome the limitations of its unstable backbone. As with the Ab responses, KLK did not overcome the limitations of the GpC ODN of either backbone (*p* > 0.05 compared to any adjuvant alone). The KPK control peptide was inactive on its own or combined with ODN for OVA-specific CD8 T cells. The formulations that induced significant levels of CD8 T cells in WT mice failed to do so in TLR9 KO mice, showing any adjuvant effects on CD8 T cells, seen in WT mice which were TLR9 dependent.

Results for OVA-specific IFNγ secretion by splenocytes paralleled exactly the CD8 T cells, namely elevated levels were seen in WT but not TLR9 KO mice and only with CpG(S) as sole adjuvant, or the combinations of KLK/CpG(S) which was not better than CpG(S) alone, or KLK/CpG(O) (*p* < 0.001). Interestingly however, this readout showed that combinations of KLK/GpC(S) induced significantly higher OVA-specific IFNγ than GpC(S) alone (Figure 2B,C). Weak to no IL-2 and IL-4 was detected in WT or TLR9−/− mice.

### 3.3. Impact of KLK on Innate Immune Stimulation

**Figure 3:**
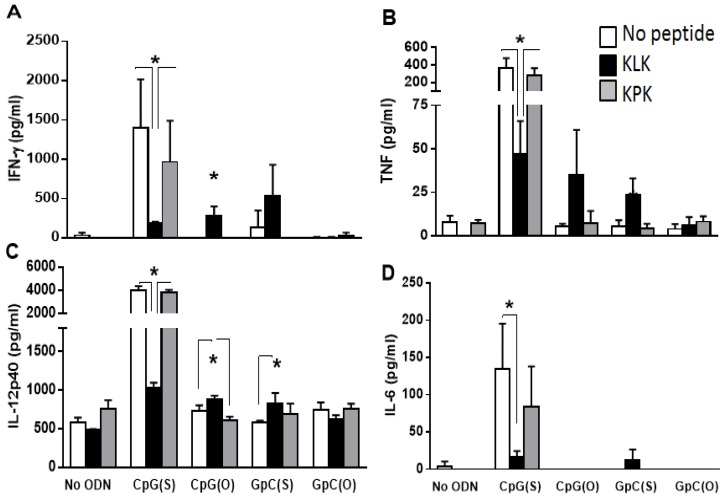
Wild type C57Bl/6 mice (*n* = 5/group) were injected with unadjuvanted OVA (10 μg) or OVA adjuvanted with CpG(S), CpG(O), GpC(S), GpC(O) ODN (4 nmol) alone or in combination with KLK peptides (100 nmol). Mice were bled at 24 h post injection plasma cytokine levels were measured by ELISA. Bars represent the group mean (±SEM). Significant differences between groups is shown by * where * = *p* < 0.05.

To help understand the adjuvant activity of KLK, we assessed its effect on innate immune responses by measuring plasma cytokines and activation of APC at 24 h after IM administration of OVA with KLK and/or ODN. As it has been well established in many studies [30,31], CpG(S) significantly enhanced plasma IFNγ, TNFα, IL12p40 and IL-6 (*p* ≤ 0.001) over background (OVA alone). In contrast, KLK, CpG(O) or GpC (in either backbone) all failed to induce plasma cytokines (Figure 3).

The KLK/CpG(S) combination was significantly worse than CpG(S) alone for induction of plasma cytokines (*p* ≤ 0.01) which was not totally unexpected since, as presented earlier, this combination trended to inferiority compared to CpG(S) alone for augmenting OVA-specific CD8 T cells. In contrast, the KPK/CpG(S) combination was not different to CpG(S) alone (*p* > 0.05) indicating that the interference seen with KLK/CpG(S) was related to the function of KLK by itself, rather than the combination of an ODN and peptide.

Combinations of KLK with CpG(O) and GpC(S) enhanced secretion of IFNγ, TNFα and IL-12p40 (*p* ≤ 0.05) compared to ODN or KLK alone but not IL-6 (*p* > 0.05). The combination of KLK with GpC(O) failed to induce cytokine secretion (*p* > 0.05) compared to OVA alone (Figure 3).

Cell activation markers measured by flow cytometry showed findings similar to the cytokine secretion. As a sole adjuvant, only CpG(S) up-regulated CD80, CD86 and MHCII on CD11b+ cells and CD86 on B cells (B220+) (*p* < 0.0001 compared to OVA alone) and the KLK/CpG(S) combination was generally worse that CpG(S) alone (*p* ≤ 0.001 for CD80 and MHC II on CD11b positive cells and *p* < 0.01 for CD86 on B cells compared to CpG(S) alone).

**Figure 4:**
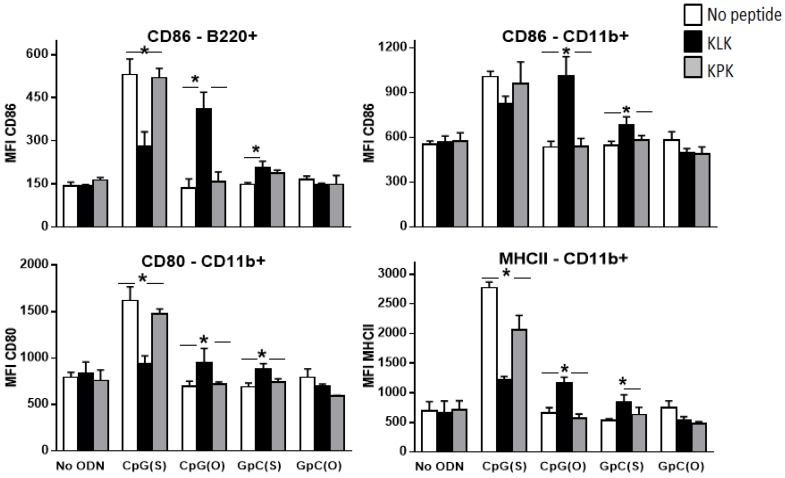
Wild type C57Bl/6 mice (*n* = 5/group) were injected with unadjuvanted OVA (10 μg) or OVA adjuvanted with CpG(S), CpG(O), GpC(S), GpC(O) ODN (4 nmol) alone or in combination with KLK peptides (100 nmol). Spleens were harvested at 24 h post injection and activation of antigen presenting cells was assessed by flow cytometry as indicated in methods. Bars represent the group mean (±SEM). Significant differences between groups is shown by * where * = *p* < 0.05.

There was some upregulation of CD86 expression on B cells (B220+) (*p* ≤ 0.01) and CD80, CD86 and MHCII on CD11b+ cells (*p* ≤ 0.01) with KLK/CpG(O) and KLK/GpC(S) compared to any of these adjuvants alone (Figure 4); KLK/GpC(O) did not activate immune cells *in vitro* (*p* > 0.05 *versus* OVA).

The above data was all in the presence of OVA, but similar data were obtained when ODN or KLK/ODN and KPK/ODN combinations were tested for innate immune activation in the absence of OVA.

### 3.4. Impact of KLK on Intracellular Delivery of CpG(S)

When administered on their own, fluorescently labeled CpG(S) was detected in association with CD11b and CD11c positive APC by 5 h post injection and this lasted for up to 48 h. Co-administration of CpG(S) with KLK delayed the first appearance of the ODN in the APC to 24 h (Table 3) and even up to 48 h MFI levels were much lower than when ODN were given alone. This data is in line with the innate and adaptive immune response data where the combination of CpG(S) and KLK was inferior to CpG(S) alone for activating innate immune responses and augmenting T cell responses.

## 4. Discussion

KLK is an anti-microbial peptide that, among other properties, can translocate into cells without cell membrane permeabilization and thus it may be useful for intracellular delivery of co-formulated compounds [4]. When mixed with an antigen, KLK on its own is a poor adjuvant. However it has previously been shown to be effective for augmenting the adjuvant effects of polyI:C, a TLR 3 agonist (as in IC31®), presumably by helping deliver the polyI:C into the endosome where TLR3 resides [25]. Herein, we show similar results in mice using KLK with ODN, which exert their adjuvant effects through activation of TLR9, also located in the endosome. The lack of adjuvant activity in TLR9 KO mice suggest that in WT mice the KLK might assists the ODN, to better reach the endosome, but possibly also to better activate TLR9 once there. Our humoral data in wild type and TLR9 KO mice confirm previous finding about ability of KLK peptide on its own to increase antibody response to antigens including ovalbumin [7]. This adjuvant effect was attributed to the fact that KLK enhances association of antigen to antigen presenting cells (APC) and forms a depot of antigen at the injection site resulting in prolonged antigen maintenance as demonstrated for Alhydrogel® (Alum) [7]. Depot effect at the injection site was observed in all animal groups treated with KLK, either alone or with ODN. No depot effect was obtained with KPK.

When CpG is combined to KLK, antibody response increased significantly, furthermore, compared to KLK, the benefit of combining KLK and an ODN was not the same for all ODN tested. GpC(O) which was inactive on its own, remained inactive when combined with KLK. Benefits of combining KLK however, was seen with CpG(O) and GpC(S) which on their own had little or no adjuvant activity. KLK enhanced all immune assay readouts and only IFN-γ secretion when combined with CpG(O) and GpG(S) respectively. In contrast, there was no benefit and in some cases it was clearly detrimental to combine KLK with CpG(S), which on its own was the most potent of all adjuvant formulations tested for both humoral and cellular immunity. The explanation for these observations comes from our current understanding how CpG ODN activates TLR9.

It was initially thought that synthetic ODN required unmethylated CpG motifs for TLR9 mediated immunostimulatory activity [25,26,32]. However, more recent results show that ODN without CpG motifs [33,34] or containing methylated CpG motifs [35] can also be immunostimulatory, albeit less potent than CpG ODN, if delivered directly to the endosome for interaction with TLR9. Both CpG and non-CpG DNA bind to TLR9 with a 1:2 stoichiometry, however the dissociation constant is lower for CpG (62 ± 9 nmol) than non-CpG (153 ± 26 nmol) DNA, which could account for the greater potency of CpG than non-CpG ODN for TLR9 activation *in vitro*, even when transfection agents are used [36]. However, in the *in vivo* situation, the absence of an adjuvant effect for ODN lacking unmethylated CpG motifs may be due more to an inability to translocate to the endosome, rather than an inability to bind to and activate TLR9 once there. The exact mechanism for endosomal entry for CpG ODN is not known, but it has recently been shown that ODN containing unmethylated CpG motifs can preferentially bind to surface membrane binding proteins, such as CD14, KIR (killer immunoglobulin receptor) or DEC-205 (a multi-lectin receptor) that are expressed on innate immune cells [37,38]. ODN without CpG motifs, such as the GpC(S) used in this study, cannot enter cells by this mechanism, but when combined with KLK, it is offered an alternate delivery method to reach the endosome where it is able to some degree activate TLR9.

The situation with KLK and CpG(O) would be somewhat different. CpG(O) contain unmethylated CpG motifs necessary for cell entry and are actually more potent than CpG(S) for *in vitro* activation of TLR9, presumably because of superior ligand binding with the non-modified backbone. However, they lack immunostimulatory activity *in vivo* due to their susceptibility to degradation by nucleases [39]. As such, the prime benefit for combining CpG(O) with KLK is likely to be enhanced protection against nucleases, possibly through the formation of KLK/ODN complexes due to ionic interactions. This statement can be verified by assessing CpG(O) integrity when incubated alone, or with KLK in the presence of serum nucleases. Similarly, CpG(O) are effective for immune stimulation or as vaccine adjuvants when delivered within a liposome or immune stimulatory complex that would provide protection from nucleases [37,40]. While the native backbone is better than a modified one for TLR9 binding, phosphorothioate backbone ODNs have been reported to increase ODN uptake by APCs [24,41,42], so the phosphorothioate backbone of CpG(S) may provide benefits beyond protection from nucleases. Hence, in addition to affording protection from nuclease-mediated destruction, KLK may also enhance cellular uptake of CpG(O).

While KLK can mitigate some of the limitations of the unstable backbone of CpG(O) and the poorly TLR9 activating sequence of GpC(S), the finding was that there was no adjuvant activity of KLK/GpC(O) over KLK alone, indicating that it cannot compensate sufficiently when both deficits appear together.

Consistent with our results, KLK peptide has been shown to deliver ODN1a in the IC31® adjuvant system into the endosomal compartment of human myeloid DC and mouse bone marrow derived DC [35]. KLK has also been shown to dramatically enhance delivery of ODN1a to human dendritic cells, whereas delivery of a CpG(S) to the same dendritic cells was not dependent on the presence of KLK [35]. Similarly, cationic lipids (*i.e.*, DOTAP) [33,34,43,44] and other antimicrobial peptides (*i.e.*, LL37) [37,45,46,47] have been shown to enhance TLR9 activation by methylated CpG and/or GpC ODN, presumably by facilitating entry into the immune cells where the ODN can access the endosome.

The finding that adjuvant effects with CpG(S) are better than those with KLK/CpG(S) may indicate that the cell entry mechanism of CpG is superior to that of KLK. However, it is also possible that it is simply a question of kinetics with the delayed and more prolonged delivery of CpG(S) when combined with KLK, resulting in poorer TLR9 activation.

## 5. Conclusions

In summary, combined data from this and other studies suggest that an ODN that contains unmethylated CpG motifs and is stable enough to survive the interstitial milieu (*i.e.*, nuclease resistant backbone) can through its own mechanisms enter immune cells and localize to the endosome, where potent activation of TLR9 results in strong adjuvant effects. KLK, likely through a different mechanism, can facilitate cell entry of ODN to access the endosome and while this is not of benefit to CpG(S), it is of benefit to ODN such as GpC(S) that lack CpG motifs to aid cellular entry. Additionally, KLK can provide essential protection from nucleases for CpG(O).

## Figures and Tables

**Table 1:** Details on oligonucleotide used.

ODN	Backbone	Sequence
CpG(S) ^1^	Phosphorothioate (PS)	TCCATGACGTTCCTGACGTT
CpG(O)	Phosphodiester (PO)	TCCATGACGTTCCTGACGTT
GpC(S)	Phosphorothioate (PS)	TCCATGAGCTTCCTGAGCTT
GpG(O)	Phosphodiester (PO)	TCCATGAGCTTCCTGAGCTT

^1^ This sequence, also known as CpG 1826, is rodent-specific B-Class CpG that has been used widely in published studies.

**Table 2:** Summary of adjuvant effects on anti-OVA antibody titers.

Compound	Peptides		Oligodeoxynucleotides (ODN)			
	KLK	KPK	CpG(S)	CpG(O)	GpC(S)	GpC(O)
Single agent	** *vs.* OVA	NS *vs.* OVA	**** *vs.* OVA	NS *vs.* OVA	NS *vs.* OVA	NS *vs.* OVA
KLK combo			**** *vs.* OVA	**** *vs.* OVA	**** *vs.* OVA	** *vs.* OVA
			NS *vs.* CpG(S)	** *vs.* KLK	NS *vs.* KLK	NS *vs.* KLK
KPK combo			**** *vs.* OVA	NS *vs.* OVA	** *vs.* OVA	NS *vs.* OVA
			NS *vs.* CpG(S)	NS *vs.* KPK	** *vs.* KPK	NS *vs.* KPK

NS not significant (*p* > 0.05); * *p* < 0.05; ** *p* < 0.01; *** *p* < 0.001; **** *p* < 0.0001; versus no adjuvant (OVA) or versus individual adjuvants for combos.

**Table 3:** Uptake of fluorescently labelled CpG(S) ODN into antigen presenting cells (APC) in draining lymph nodes at various times after *in vivo* delivery by IM injection.

Treatment	CD11c+ APC			CD11b+ APC		
	5 h	24 h	48 h	5 h	24 h	48 h
Naïve (no ODN)	121 ± 2	121 ± 2	121 ± 2	99 ± 8	99 ± 8	99 ± 8
CpG(S)	409 ± 45	584 ± 105	565 ± 71	384 ± 30	405 ± 6	324 ± 1
CpG(S) + KLK	124 ± 6	133 ± 4	157 ± 8	92 ± 9	109 ± 10	117 ± 1

Numbers correspond to mean Mean Fluorescence Intensity, MFI (±SEM).

## Abbreviations

The following abbreviations are used in this manuscript:

TLR	Toll Like Receptor
APC	Antigen Presenting Cell
ODN	Oligideoxynucleotide
ELISpot	Enzyme-Linked Immunospot
ELISA	Enzyme-Linked Immunosorbent Assay
WT	Wild Type
FAM	Fluorescein Amidite
OVA	Ovalbumin
DC	Dendritic Cell
